# Human Oligodendrocytes and Myelin In Vitro to Evaluate Developmental Neurotoxicity

**DOI:** 10.3390/ijms22157929

**Published:** 2021-07-25

**Authors:** Megan Chesnut, Thomas Hartung, Helena Hogberg, David Pamies

**Affiliations:** 1 Center for Alternatives to Animal Testing (CAAT), Johns Hopkins Bloomberg School of Public Health, Baltimore, MD 21205, USA; meganechesnut@gmail.com (M.C.); thartun1@jhu.edu (T.H.); 2 Center for Alternatives to Animal Testing (CAAT-Europe), University of Konstanz, 78464 Konstanz, Germany; 3 Department of Physiology, University of Lausanne, 1005 Lausanne, Switzerland; 4 Swiss Centre for Applied Human Toxicology (SCAHT), 4055 Basel, Switzerland

**Keywords:** developmental neurotoxicity, neurotoxicity, organotypic, organoid, myelin, developmental diseases, oligodendrocytes

## Abstract

Neurodevelopment is uniquely sensitive to toxic insults and there are concerns that environmental chemicals are contributing to widespread subclinical developmental neurotoxicity (DNT). Increased DNT evaluation is needed due to the lack of such information for most chemicals in common use, but in vivo studies recommended in regulatory guidelines are not practical for the large-scale screening of potential DNT chemicals. It is widely acknowledged that developmental neurotoxicity is a consequence of disruptions to basic processes in neurodevelopment and that testing strategies using human cell-based in vitro systems that mimic these processes could aid in prioritizing chemicals with DNT potential. Myelination is a fundamental process in neurodevelopment that should be included in a DNT testing strategy, but there are very few in vitro models of myelination. Thus, there is a need to establish an in vitro myelination assay for DNT. Here, we summarize the routes of myelin toxicity and the known models to study this particular endpoint.

## 1. Introduction: Role of Environmental Factors in an Epidemic of Adverse Neurodevelopmental Outcomes

One in every six children in the United States, or about ten million children, were diagnosed with a developmental disorder from 2006 to 2008, according to the latest report by the Centers for Disease Control and Prevention (CDC) National Center on Birth Defects and Developmental Disabilities (NCBDDD) [1]. This represents an increase in prevalence of about 17% in the 12 year period from 1997 to 2008, or nearly 1.8 million more children with at least one developmental disability from 2006 to 2008 compared to the previous decade [1]. It was reported that this trend was primarily due to an increase in the prevalence of neurodevelopmental disorders [1]. Children with neurodevelopmental disorders can exhibit a wide range of functional deficits, including: learning disabilities; limited control of executive functions; sensory impairments; language, social, or motor limitations; and an inability to achieve expected milestones in development [2].

In recent decades, the prevalence of certain neurodevelopmental disorders, such as autism spectrum disorder (ASD) and attention deficit/hyperactivity disorder (ADHD), has increased considerably [1,2,3,4,5,6,7]. The CDC Autism and Developmental Disabilities Monitoring (ADDM) Network estimates that one in 54 children has been identified with ASD [8], and the NCBDDD of the CDC reports that 9.4% (6.1 million) of children in the United States were diagnosed with ADHD in 2016 [9]. Although an increase in prevalence could be influenced by several other factors, including the increased awareness and recognition of symptoms or behavioral phenotypes, improved diagnostic capabilities, changes in the definitions of conditions, better reporting, older parental age, or medical advances that allow increased survival rates of preterm infants with borderline viability [1,10], increased exposure to environmental contaminants is thought to play a substantial role [11,12,13].

Despite intense research, the exact etiologies of most neurodevelopmental disorders remain undetermined [11,12,13,14]. Although many neurodevelopmental disorders are thought to have a genetic basis, there is increasing evidence of an environmental influence [15,16,17], as demonstrated in monozygotic twin studies [18,19,20]. Overall, it has been estimated that about 3% of developmental defects are directly attributable to maternal exposure to chemical or physical agents in the environment, and approximately 25% are due to gene–environment interactions in genetically predisposed individuals (NRC 2000). However, these rough approximations are reported to likely underestimate the magnitude of the environmental influence on neurodevelopment [21].

Furthermore, there are concerns that environmental chemicals are contributing to a subclinical pandemic of neurodevelopmental toxicity, with reductions in cognitive function characterized by a slight decrease in intelligence quotient (IQ). These diseases might even be more prevalent than neurodevelopmental disorders with clinically observable behavioral phenotypes (such as ASD and ADHD) [11]. Although it is difficult to determine that prior exposure to a toxic agent during neurodevelopment is the cause of lowered IQ [22], this has been documented as a result of lead exposure, in which an inverse relationship was identified between blood-lead level and IQ score [23]. Such outcomes have significant repercussions on society as a whole [22]. It has been estimated that a relatively small drop (five points) in the average IQ of a cohort of lead-exposed children greatly affects the IQ distribution in a population, resulting in a more than 50% decrease in the number of gifted individuals (IQ > 130) and more than 50% increase in the number of individuals with significant cognitive impairment (IQ < 70) [22,24]. Given the potential societal implications of reduced cognitive function resulting from childhood exposures, the environmental influence on neurodevelopment is a major public health concern, particularly because children are a uniquely susceptible population [11].

### 1.1. Pre- and Postnatal Susceptibility to Environmental Exposure

Compared to adults, infants and children have a higher air, food, and water intake relative to their body weight, and, consequently, they are exposed to higher doses of environmental contaminants [24,25,26]. In addition, their immature metabolism increases their susceptibility to adverse effects following exposure to a toxic agent [12,27]. Postnatal exposure to xenobiotics can also occur during breastfeeding; lipophilic compounds stored in maternal adipose tissue are mobilized with fat in breast milk production and can diffuse into the mammary glands and be excreted during lactation [28,29]. In addition, the unique activities of children, such as hand-to-mouth behavior and crawling or playing close to the ground, also make children particularly susceptible to environmental exposure [26,27].

Although the susceptibility of infants and children has been long recognized, prenatal vulnerability to environmental exposure was not acknowledged until the causal association was made between birth defects and thalidomide (a drug that had been prescribed to pregnant women for morning sickness) [21]. Prior to the recognition of birth defects attributed to thalidomide in the 1960s, chemicals were not routinely tested for their teratogenicity, due to the assumption that the placental barrier completely protected the fetus from toxicant exposure [21]. The placenta provides the exchange of nutrients, oxygen, and fetal waste products between the fetal and maternal circulatory systems, and the placental barrier describes the interface at which this exchange occurs [30]. The placenta is not completely protective, because the transplacental passage of xenobiotics and their metabolites across the placental barrier can occur [28]. The ability of a compound to cross the placental barrier depends on its physical and chemical properties, and the mechanisms of exchange across the placental barrier include the passive diffusion of lipophilic compounds, facilitated diffusion by transporters, endocytosis, and active transport [30,31]. Compounds can also be metabolized by the placenta, as it is capable of both phase I and phase II biotransformation, which can alter transplacental passage [30]. In addition, polar metabolites formed in the fetal compartment can accumulate, as their lipophobicity may prevent transport across the placental barrier [32].

The blood–brain barrier (BBB) permeability to xenobiotics or their metabolites is also an important factor in pre- and postnatal susceptibility. The BBB separates the central nervous system (CNS) from the bloodstream [28,33,34], and the capillaries that form the BBB are comprised of tight junctions of adjacent endothelial cells, effectively preventing the paracellular transport of hydrophilic compounds that can occur in other organs [28], thus protecting the brain from many neurotoxicants [33,35]. The endothelial cells of the BBB also contain efflux transporters, which actively transport certain nonpolar compounds across endothelial cell membranes back into the blood to prevent entry by diffusion into the brain [28]. Although the BBB is protective, lipophilic compounds may still bypass efflux transporters to cross endothelial cell plasma membranes, and certain polar compounds may be actively transported across the BBB [28,36]. In addition, the BBB is not established until about 23–32 weeks of gestation [37,38], and thus, the central nervous system is less protected from hydrophilic compounds in the very early stages of development, during which time the developing brain is particularly vulnerable.

### 1.2. Vulnerability of the Developing Brain to Environmental Exposures

Human brain development involves complex molecular, cellular, and environmental interactions that begin during the first month of gestation [39,40,41,42]. Due to neural plasticity—the adaptability of the brain—the developing human brain is uniquely vulnerable to toxic insult [42,43]. Moreover, nervous system development is progressive, meaning that emerging structures provide the basis for further development [42]. Considering this progression and adaptability, there exist critical windows in embryonic and fetal development in which the consequences of inhibited or altered neurodevelopment can be permanent, and there is often a delay between chemical exposure during development and an adverse neurologic outcome [12,34,41,43,44,45]. Furthermore, even minor changes in brain development can result in a dramatic loss of cognitive function [34]. Consequently, developmental neurotoxicity (DNT) is distinct from adult neurotoxicity [12]. DNT can manifest in many other forms, including differences in cell type proportions, altered cell migration and organization, functional changes, or a lack of network connectivity [39]. In addition, DNT often occurs at doses lower than those, which cause adult neurotoxicity and transient insults that result in reversible effects in adults (and can cause irreversible damage during neurodevelopment) [12].

## 2. Limited Current Knowledge of Chemicals That Disrupt Neurodevelopment

DNT is among the least studied forms of toxicology [46,47], and most substances in commercial use today, even of those classified as high-production-volume chemicals, have not been screened for DNT [15,48,49,50]. Traditionally, DNT has only been studied following a poisoning incident or after neurotoxicity was observed in adults [15], and regulatory action often lags far behind the first recognition of DNT [24]. Two classic examples of developmental neurotoxicants are lead and methylmercury, and it took about 25 years for lead to be recognized as a developmental neurotoxicant and removed from gasoline [51]. About 50 years after the first report of methylmercury-induced neurodevelopmental effects, it was finally considered in risk assessment [52].

A scope review conducted by Grandjean and Landrigan (2006) described the extent of the current knowledge of chemicals that disrupt neurodevelopment as “the tip of a very large iceberg” [15]. This review identified only five compounds that had been convincingly demonstrated to cause DNT in humans [15]. The five DNT compounds included lead, methylmercury, polychlorinated biphenyls (PCBs), arsenic, and toluene [15]. This list did not include ethanol, as maternal exposure to ethanol was considered voluntary [15]. The list of DNT compounds was later expanded to include tetrachloroethylene, manganese, chlorpyrifos, brominated diphenyl ethers (BDEs), dichlorodiphenyltrichloroethane (DDT), fluoride, and dichlorodiphenyldichloroethylene (DDE) [11], and recent clinical studies have suggested the addition of valproic acid to the list of known DNT compounds [44,46,53]. Thus, only 14 chemicals are known to be toxic to human neurodevelopment [11,15,44,46,53]. This number is likely not representative of the actual number of compounds that cause DNT, emphasizing the need for improved methods of DNT evaluation to expand current knowledge and inform regulatory decision making [15]. In addition, as few chemicals are classified as being developmentally neurotoxic, it is also difficult to establish reference compounds to evaluate the specific DNT assays. However, suggestions of criteria to select reference compounds for developmental neurotoxicity (DNT) have been published recently [44]. The authors of this review convened in a workshop to define criteria for the selection of positive and negative reference compounds [44].

### 2.1. Constraints of Traditional Developmental Neurotoxicity Testing

Regulation focused on protecting neurodevelopment is restricted by current methods in regulatory developmental neurotoxicity testing [11,12,15]. Current DNT guidelines from both the US (USEPA 712-C-98-155 239) and internationally (OECD TG 426) recommend the use of animal testing with rodents to classify developmental neurotoxicants [54,55]. To meet the requirements of both the Federal Insecticide, Fungicide, and Rodenticide Act (FIFRA) and the Toxic Substances Control Act (TSCA), which was recently reauthorized as the Frank Lautenberg Chemical Safety for the 21st Century Act in 2016, the US EPA recommends exposure at three doses with a control and several groups of pregnant rats exposed during gestation and early lactation. At least 20 litters for each dose level are recommended, along with the evaluation of maternal toxicity and DNT evaluation based on a series of tests to examine motor activity, auditory startle, learning and memory, brain weight, and neuropathology in offspring through adulthood [54]. Similarly, the OECD advises the use of rats, 20 litters of per dose level, and the observation of gross neurologic and behavioral abnormalities with evaluations of physical development, behavioral ontogeny, motor activity, motor and sensory function, learning and memory, brain weight, and neuropathology during postnatal development and adulthood [55]. These testing methods are time-consuming, resource-intensive, and expensive [12]. It is estimated that a guideline DNT study for one chemical takes about 3 months, costs approximately USD 1.4 million, and requires a minimum of 140 mated female rats to produce 1000 rat pups [12]. It is therefore not feasible to screen the thousands of untested chemicals with potential to cause DNT using in vivo guideline studies [12].

Moreover, there are concerns regarding the physiological relevance of extrapolating the results from animal studies to human health effects [12,56,57]. There is evidence of considerable disagreement between the animal and human response to toxic agents [56]. The repercussions of interspecies differences are most evident in clinical trials for drugs [58], in which about 92% of substances fail—often due to effects in humans that were not observed in preclinical animal studies [59]. The human and rodent brain also differ considerably [60,61]. Despite the evolutionary conservation of genes important to neurodevelopment across species, gene expression during development is not conserved between rodents and humans [62]. Furthermore, studies conducted to ascertain differential developmental neurotoxicity comparing the response of rodent and human neural progenitor cells in vitro have elucidated species-specific effects of compounds on key neurodevelopmental events, such as proliferation, migration, and differentiation [63,64].

### 2.2. Opportunities for Integrated Developmental Neurotoxicity Testing Strategies

The use of alternative methods in toxicology, either as replacements or supplements to animal testing, will allow more cost-effective and human-relevant chemical screening for DNT [49,65]. Toxicology is currently experiencing a paradigm shift from the reliance on observational animal experimentation to mechanism-based science, which supports this concept [56,66,67,68,69]. The paradigm shift is outlined in the 2007 U.S. National 188 Research Council (NRC) report Toxicity Testing in the 21st Century: A Vision and a Strategy, in which the NRC Committee on Toxicity Testing and Assessment of Environmental Agents recommended that toxicity testing should focus on the mechanistic evaluation of toxicity pathways through the combined use of in vitro and in silico technologies, with less reliance on conventional in vivo animal experiments [70]. This transition to the use of alternatives to animal testing is also supported by recent regulatory efforts, such as the EU Registration, Evaluation, Authorisation and Restriction of Chemicals (REACH) of June 2007, which recommended the replacement, reduction, and refinement of animal testing, as well as the Frank R. Lautenberg Chemical Safety for the 21st Century Act, which amended the Toxic Substances Control Act (TSCA) in June of 2016 in the US and recommended methods that reduce or replace the use of vertebrate animals1. Thus, the development of novel alternative methods is considered the “future of toxicology” [69]. An important consideration, however, is that a single assay does not provide sufficient information to replace an animal model or facilitate regulatory decision making, and, as a result, current efforts are focused on the development of integrated testing strategies that systematically combine information from multiple sources and test systems to quickly and reliably identify toxicity [71].

It is widely acknowledged that DNT is a consequence of disruption to the fundamental processes that define neurodevelopment at the cellular level [12,46,72,73]. A comprehensive in vitro developmental neurotoxicity testing strategy should, therefore, consider each of these processes, which include proliferation, migration, differentiation, synaptogenesis, myelination, apoptosis, and the formation of functional neuronal networks [28,39,40,41,42,65]. The ultimate goals of these new methods are their usage for regulatory purposes [74]. OECD/EFSA have recently started a program with the consensus of the DNT community after several workshops [75] and meetings to develop a testing battery for DNT, in which the aim is to present enough data and outline what could become an integrated approach to testing and assessment (IATA) for the purposes of screening and prioritization or hazard assessment [75]. Moreover, with regard to the federal insecticide, fungicide, and rodenticide act (FIFRA), the US EPA recently had a public virtual peer review meeting for the use of new approach methodologies (NAMs) to evaluate DNT for human risk assessment. Among the NAMs discussed were two assays performed by the EPA and the whole testing battery evaluated by EFSA [76] (see above). The overall response by the scientific advisory panel to NAMs to DNT was positive. However, one of the main criticisms was the lack of assays in the battery addressing glial toxicity, including myelination.

## 3. Myelination as a Developmental Neurotoxicity Endpoint

### 3.1. Oligodendrocyte Differentiation, Maturation, and Myelination

Myelination is a particularly important endpoint to consider in a DNT testing strategy due to the significant involvement of the process in the maturation and function of the human brain [77,78,79]. Myelination begins around the fifth month of fetal development but is prolonged through adolescence [80,81,82]. A peak in myelination rate occurs at 2–3 years of age [83], but ongoing myelination and increased white matter volume continue in children aged 12–18 years [84,85]. Around age 20 and throughout life, gray matter volume starts to decline [86,87]. The formation of myelin begins with the specification of oligodendrocyte precursor cells (OPCs) from neural progenitor cells (NPCs) [79,88,89]. OPCs then migrate to their target axons, differentiate into pre-oligodendrocytes (pre-OLs), then differentiate into pre-myelinating oligodendrocytes (pre-myelinating OLs), and finally differentiate into mature and/or myelinating oligodendrocytes (OLs) [79,88,89]. The differentiation of oligodendrocytes following axogenesis is believed to be mediated by complex trophic signals between the oligodendrocytes and neurons. During differentiation into mature oligodendrocytes, the morphology changes to a large network of branching processes [90].

### 3.2. Functions of Oligodendrocytes and Myelin

The myelination of axons in the central nervous system (CNS) is crucial for brain function [77] and is one of the key events in brain development. Myelin is formed from the processes of oligodendrocytes that provide electrical insulation and accelerate transmissions along the axon [91]. In the CNS, one oligodendrocyte can produce about 100 myelin sheaths for several different neurons [92]. The sheaths are formed by multiple alternating protein–lipid layers with a characteristic composition different from other cellular membranes [93]. It consists of 70–85% lipids (mainly phospholipids, glycolipids, and cholesterol) and 20–30% proteins, such as myelin basic protein (MBP), proteolipid protein (PLP), 2′3′-cyclic-nucleotide 3′-phospodiesterase (CNP), myelin-associated glycoprotein (MAG), and myelin oligodendrocyte glycoprotein (MOG) [93,94,95,96]. The myelin sheaths along the axons are separated by gaps exposed to the extracellular space, referred to as nodes of Ranvier, which are highly enriched in ion channels and mediate the exchange of ions during the action potential [77,79,97,98]. Propagation of the action potential between the nodes in myelinated axons (saltatory conduction) is much faster than in unmyelinated axons [95]. The increase in action potential speed reduces the need for the ATP-dependent sodium–potassium exchange required for the maintenance of the resting membrane potential along axons [89]. However, oligodendrocytes have other important roles during development and in the mature brain, such as the regulation of neurotransmitter release and metabolism [99,100], synaptic plasticity [101,102,103], axonal conductance and synaptic efficacy [99,100], neuronal signaling networks [104,105,106], neuronal excitability [107], and axonal growth, metabolism, integrity, and survival [97,108,109,110,111,112,113]. Therefore, the failure to form or maintain myelin can disrupt neuronal signal transmission or trigger the degradation of axons, and a reduction in the velocity of action potentials can lead to physical or mental disability and induce severe neurological symptoms [79]. Myelin development is also associated with improved language, memory [114], motor-skill learning [115,116], and reading performance in childhood [117]. Animal studies have implicated white matter deficits during critical periods of development in learning and memory impairments [118,119] and in an inability to learn motor skills [116]. Moreover, there is evidence for oligodendrocyte and myelin dysfunction in 258 neurodevelopmental disorders with cognitive symptoms (e.g., Williams syndrome, Pitt-Hopkins syndrome, and autism) [120,121].

## 4. Potential Mechanisms of Toxicity to Oligodendrocytes and Myelin

**Figure 1:**
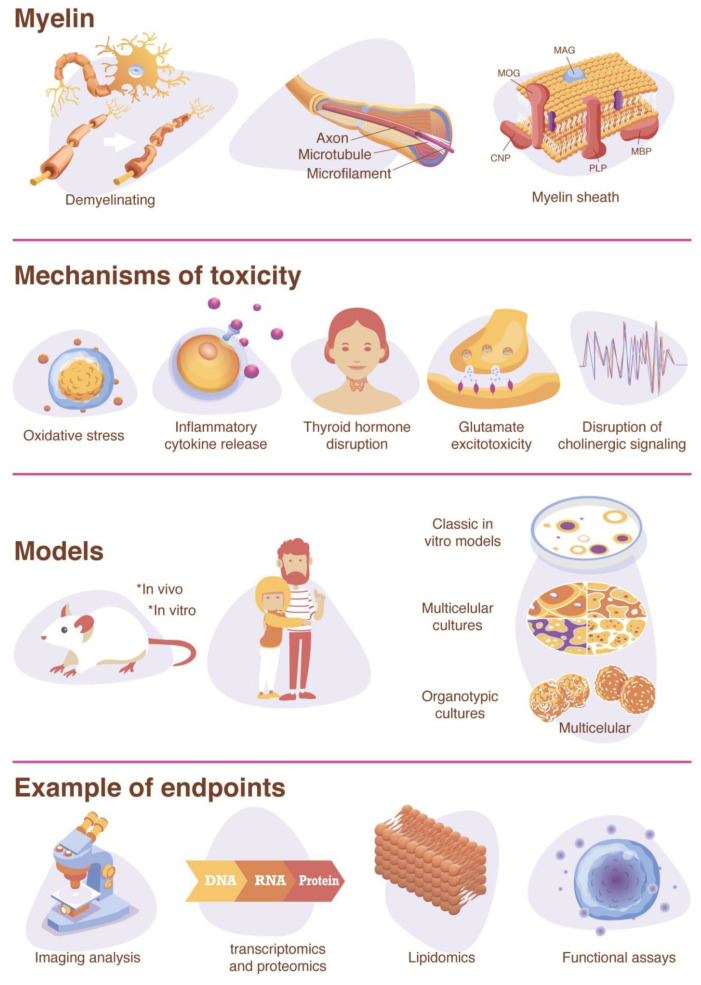
Graphical summary.

As oligodendrocytes are operating near their metabolic capacity, they are especially sensitive to chemical disturbances and alternations in oligodendrocyte proliferation [122]. Chemicals proposed to have an effect on the myelination process include ethanol [123], tellurium [124,125,126], Bisphenol A (BPA) [127,128,129], TDCPP [130], Cuprizone [131], sodium metavanadate [132], methylmercury [133], and lead [134,135,136,137,138]. However, there is limited information on the mechanism involved, and only few chemicals have been identified to include DNT. Possible mechanisms of toxicity to oligodendrocyte differentiation include the inflammatory cytokine release by microglia [139], thyroid hormone disruption [65], glutamate excitotoxicity [140,141,142], as well as the disruption of cholinergic signaling (e.g., acetylcholinesterase inhibition) [141,143] (Figure 1). Many of these mechanisms involve toxicity pathways that ultimately result in cell death by oxidative stress [139,144].

### 4.1. Inflammatory Cytokine Release

Following systemic infection, inflammation, or neural damage, microglia in the brain can become activated and release inflammatory cytokines [139], such as interleukins (IL-1 and IL-6), tumor necrosis factor alpha (TNF-α), interferon gamma (IFN-γ), and transforming growth factor beta (TGFβ). These cytokines have been shown to induce white matter lesions in the CNS [145]. Maternal immune activation has also been shown to alter myelination during neurodevelopment [146,147], and the proinflammatory cytokines IL-6, IL-1, and TNF-α are thought to play roles in prenatal white matter damage that occurs following maternal intrauterine infection [148]. In addition, the prenatal exposure to lipopolysaccharide (LPS), a bacterial endotoxin that elicits an immune response [28], has been shown to alter myelination in rats [149].

### 4.2. Thyroid Hormone Disruption

Alterations of thyroid hormone homeostasis during this time span might affect the myelination process and cause persistent adverse outcomes [150]. There is strong evidence demonstrating that thyroid hormone (TH) signaling is integral to myelin formation because TH is required for the regulation of OL differentiation and maturation [151,152,153,154,155,156,157]. Moreover, there is evidence that environmental chemicals that disrupt thyroid hormone function also disrupt myelin formation [158,159,160,161]. For example, the polybrominated diphenyl ether (PBDE) flame retardant BDE-99 was recently demonstrated to inhibit NPC differentiation into the OL lineage in vitro [162], and PBDE flame retardants are known thyroid hormone disruptors [163]. In addition, the leading commercial pentaBDE technical mixture, DE-71, which contains BDE-99 [164], has also been shown to downregulate the gene expression of myelin basic protein in exposed zebrafish embryos [158,160].

### 4.3. Glutamate Excitotoxicity

Glutamate excitotoxicity from the sustained activation of OL glutamate receptors can lead to oxidative stress in oligodendrocytes through an influx of calcium and subsequent production of reactive oxygen species (ROS) [140,142,165]. Because the calcium influx occurs through calcium-permeable kainate and α-amino-3-hydroxyl-5-methyl-4-isoxazole-propionate (AMPA) receptors, which are expressed at higher levels in developing OLs [140,166], they are more vulnerable to excitotoxicity than mature OLs are [140,142]. Excitotoxicity can also be mediated by other cell types in the brain [139,167]. For example, astrocytes regulate glutamate homeostasis through glutamate uptake [167] and activated microglia can generate ROS and reactive nitrogen species (RNS), which reduce the expression of glutamate transporters and prevent glutamate uptake. OLs are more susceptible to ROS (see below), which can contribute to increased OL susceptibility to glutamate excitotoxicity [139,167].

### 4.4. Disruption of Cholinergic Signaling

Oligodendrocytes express muscarinic and nicotinic acetylcholine (ACh) receptors [103,168,169,170,171,172], and acetylcholine signaling is thought to play a role in the production and maintenance of myelin [103]. An environmental compound that acts as an agonist for the nicotinic ACh receptors is nicotine. Exposure to nicotine has been shown to affect oligodendrocyte maturation and myelin formation [103], and maternal nicotine exposure has been demonstrated to alter myelin-related gene expression in adolescent rats [173] and zebrafish offspring [174].

Inhibitors of acetylcholinesterase (AChE), the enzyme that hydrolyzes acetylcholine to acetate and choline, have been shown to inhibit myelin formation as well [103]. Environmental AChE inhibitors include organophosphates or carbamates nerve agents and insecticides, and any axoglial communication required for myelin formation that relies on cholinergic signaling could potentially be affected by AChE inhibitors [103]. Chlorpyrifos, an organophosphate insecticide known to inhibit AChE, is thought to be associated with autism risk [175] and has shown DNT effects on oligodendrocyte differentiation and myelin formation at concentrations below those that cause systemic toxicity due to AChE inhibition [176,177]. The US EPA has updated its Human Health Risk Assessment in 2016 to reflect the possibility of toxicity at exposures below levels that cause 10% red blood cell AChE inhibition—the level that was used previously to predict DNT [175]. Notably, organophosphate flame retardants (OPFRs), while structurally similar to organophosphate pesticides, do not as effectively inhibit acetylcholinesterase [178,179]. However, OPFRs have still been demonstrated to elicit DNT effects on oligodendrocytes, such as 1,3-dichloro 2-propyl phosphate (TDCPP), which has been studied in zebrafish embryos and is thought to act through thyroid hormone disruption [130]. Furthermore, several OPFRs affected the expression of genes involved in myelination in a 3D rat primary in vitro model [180].

### 4.5. Oxidative Stress

Oligodendrocytes are known to be sensitive to oxidative stress caused by reactive oxygen and nitrogen species (ROS and RNS, respectively) [90,140,181]. ROS are produced by oxidative phosphorylation, and a main source is the mitochondrial electron transport chain [182,183]. At very low levels, ROS and RNS act as second messenger signaling molecules [184]; however, when levels exceed antioxidant levels, this can result in mitochondrial dysfunction and oxidative stress [182]. The level of sensitivity to oxidative stress depends on the OL differentiation stage [144,185]. Oligodendrocyte precursor cells (OPCs) are particularly susceptible to oxidative stress because they have lower levels of antioxidants, such as glutathione, and higher levels of iron compared to mature OLs [140,185]. The maturation-dependent susceptibility to oxidative stress has been demonstrated by depleting intracellular glutathione, a regulator of intrinsic antioxidant protection, in OLs at various differentiation stages in vitro, demonstrating that mature OLs were less vulnerable to cell death caused by oxidative stress than their precursors were [144].

### 4.6. Disruption of Ion Channel Signaling

Myelin isolates the axons of neurons to favor a more efficient action potential propagation by saltatory conduction. This process requires a high density of voltage-gated channels in the areas without myelin (called the nodes of Ranvier) [186]. These specialized axonal domains differ dramatically from internodal axonal regions, with high density in voltage-dependent Na1 (Nav) channels, to juxtaparanodal domains, where Kv channels are clustered beneath the myelin sheath [187]. The lack of oligodendroglial signals observed in neuroinflammatory disorders, such as multiple sclerosis (MS) or in experimental animal models of demyelination, has shown the reorganization of voltage-gated ion channels to compensate the saltatory loss. Animal studies have suggested that changes in the subunit expression and redistribution of these channels can follow traumatic nerve injury [188]. In addition, brain disorders associated with neuronal excitability, such as epilepsy, are linked to Nav channel alterations, suggesting the importance of this structure [189].

Ca^2+^ activity is also very important during myelin sheath formation and growth [190]. In vivo studies have indicated that local Ca^2+^ signaling regulates distinct stages of myelination [191]. In vitro studies have shown how compounds such as BDE-47 and 6-OH-BDE-47 interfere with Ca^2+^ signaling during development [192] and can alter oligodendrocytes differentiation [193].

## 5. Existing NAMs Used to Study Toxicity to Oligodendrocytes and Myelination

There are several existing methods for evaluating oligodendrocyte toxicity in vitro, with pure cultures of OPCs or OLs and corresponding assays for proliferation, migration, and differentiation, and this has been reviewed elsewhere [194]. In addition, new protocols to generate oligodendrocytes from human iPSCs have been developed [195,196]. However, there are challenges to culturing these cells for a longer period of time, and they also lack the maturation diversity and cytoarchitecture that oligodendrocytes present in vivo. The central limitation of pure OPC/OL culture systems is that cell–matrix or cell–cell interactions between oligodendrocytes and neurons or other glial cells cannot be studied [194]. To observe these interactions, methods of co-culturing neurons and oligodendrocytes have been developed [197,198,199]. Still, these systems do not incorporate other types of glial cells and most are cultured under 2D conditions [194]. The majority of in vitro models of oligodendrocytes and myelination that comprise more than neurons and OLs are derived from rodent primary or stem cells [65], including ex vivo rodent brain slice cultures [200,201,202], 3D organotypic cultures [203,204,205,206,207], and microfluidic cell culture systems [208]. However, the study of myelin formation during neurodevelopment warrants human cell-based DNT testing methods due to species differences. Differential gene expression analysis has revealed 244 genes expressed in human OPCs that are not expressed in their mouse counterparts [209]. In addition, the protein composition of myelin is not fully conserved across species [210]. A 3D model derived from primary fetal human neural progenitor cells (hNPCs) has been successfully used to determine the effect of DNT compounds in oligodendrocyte differentiation [193], showing the advantages of 3D cultures for studying oligodendrocyte maturation. Unfortunately, very few in vitro human cell-based organotypic models of myelination exist. This is partly because there are limited protocols available to efficiently myelinate axons in vitro, and sufficient myelination by oligodendrocytes typically requires in vivo transplantation in animal models [211].

### Organotypic Human Models to Study Myelin

Only a few human 3D models have been reported to include myelination by oligodendrocytes. Most of these models are generated from iPSCs; human embryonic stem cells, however, have also been used. Sandstrom and collaborators have developed a human embryonic stem cell-derived 3D neural tissue model of neurons, astrocytes, and oligodendrocytes in which immature myelin sheaths are formed around axons after two months in culture [212]. In the case of iPSC-derived organoids, different approaches have been applied to achieve myelination. For example, Madhavan and collaborators used iPSC-derived spheroids (oligocortical spheroids; OCS) to achieve myelination. The OCS generated robust populations of oligodendrocytes across different cell lines, showing expressions of proteolipid protein 1 (PLP1) (oligodendrocyte membrane protein) and MYRF25, a transcription factor specifically expressed in oligodendrocytes in the CNS. After 20 weeks in culture, they were able to observe myelin compaction [213]. Furthermore, Kim and collaborators generated fused forebrain organoids (FFOs) by combining ventral forebrain organoids (VFOs) with dorsal forebrain organoids (DFOs) that were able to promote oligodendroglia maturation in 12 weeks [214]. This model was extensively characterized, with compact myelination produced by the present oligodendrocytes. The oligodendroglia in this study seems to be dorsally derived, and the authors believed that the observed processes recapitulate the third wave of oligodendrogenesis [214]. Furthermore, Pamies and collaborators were able to generate a large number of myelinated axons (between 40 and 50% of total axons) using a simpler and faster protocol. In only 8 weeks, these iPSC-derived organotypic models (BrainSpheres) were differentiated into approximately 350 μm spheroids consisting of different cell types that facilitated cell–cell interactions and neuron–glial function (such as myelination) [215]. An increase in myelination as the system matured was confirmed with confocal imaging and electron microscopy that showed multiple layers of myelin wrapping the axonal structures [215]. Similarly, Pasca and collaborators used their human iPSC-derived model (human oligodendrocyte spheroids; hOLS) to generate oligodendrocytes. They claimed that in 37 days, they could generate transcriptionally similar stages to human primary oligodendrocytes [216]. Another approach has been to generate differentiated oligodendrocytes in a monolayer (70% of O4+ oligodendrocytes) within 28 days and then introduce them to 3D nanofiber scaffolds with neurons [196]. In addition, the quantification of this endpoint is crucial for DNT and NT assessment. To this point, only two models have shown the ability to quantify myelin [217,218]. Furthermore, Chesnut and collaborators were able to determine four reference compounds for chemical-induced myelin disruption, and they evaluated them with several myelin quantification endpoints [218].

The advantages of these models are that iPSCs can be used to explore sensitivities associated with different genetic backgrounds [14,34], and their differentiation into neural cell lineages is comparable to brain development in utero [14,219]. However, it should be acknowledged that more complex models bring some limitations and disadvantages compared to in vivo studies and simpler in vitro systems. Some of these limitations and disadvantages have been summarized elsewhere [220,221] and include, e.g., lower reproducibility, in many cases, the lack of immune cells and hormone systems, and the lack of vasculature and organ–organ interactions. Nevertheless, they have the potential to surpass animal models as they more closely mimic human physiology.

## 6. Endpoints to Study Toxicity of Oligodendrocytes and Myelination

Cytotoxicity assays (such as resazurin, LDH, and MTT) are used extensively in toxicology to evaluate cytotoxicity. However, they cannot be utilized to determine specific toxicity, such as neuro or oligodendrocyte toxicity, in heterogeneous cell models. Toxicity to different cell phenotypes can instead give an idea of the compound’s specificity and provide information about the molecular mechanism of action. For this reason, methods that discriminate from general toxicity effects to a more functional or structural method are preferable, especially in developmental neurotoxicity, where effects are generally below cytotoxic levels (Figure 1).

Several markers associated with oligodendrocytes and myelin can be studied by different techniques to assess glia toxicity. Commonly used oligodendroglia lineage developmental markers have been summarized by Deng and Poretz in a previously published review [90]. These markers cover immature to mature oligodendroglia stages and include maturation and differentiation. These markers can be used to quantify oligodendrocytes and myelin with techniques such as image analysis combined with immunohistochemistry and flow cytometry. In image analysis, pictures can be obtained manually [99] or by using new high-throughput imaging instruments (such as Aperio T2 ScanScope, MicroMatrices microTMA, or PerkinElmer Opera Phoenix) [222,223]. Many of these high-throughput imaging technologies have been summarized elsewhere [224]. Oligodendrocytes can then be counted manually [225] or by image analysis tools (such as simple total fluorescent quantification), more complex methods (such as semi-automated computer platforms) [208], or very sophisticated single-cell resolution quantification [226]. Flow cytometry has also been applied to quantify oligodendrocytes [196]; this technique, however, may be difficult for 3D multicellular models, as disaggregation to single cells can damage the cells.

The same markers can also be studied by gene expression analysis [180]. For example, the gene expressions of different myelin and oligodendrocyte makers were assessed in BrainSpheres after the exposure to different demyelinating compounds [218]. However, gene expression changes could be temporary and do not necessarily lead to adverse outcomes. For this reason, it is important to combine gene expression with more functional assays, such as protein analysis or morphological evaluations.

Other, more specific endpoints can be measured to understand the molecular mechanism of certain compounds (e.g., specific growth factors and cytokines that regulate oligodendrocytes, neurotransmitters, glutamate excitotoxicity, and oxidative stress). However, these endpoints can be challenging and labor-intensive to study in multicellular models, either in vivo or in vitro, where cell–cell and organ–organ interactions can contribute or protect the cells from the compound’s target mechanism. The use of 3D models in which the cellular density is higher than in traditional monolayer cultures complicates single-cell evaluation even more. To determine cell-specific effects, the use of a homogenous cell population would be advantageous. However, this would reduce the complexity and physiological relevance, as the cell–cell interactions are of high importance for the function of an organ, especially the brain. New technologies are emerging with higher resolution (single-cell resolution), allowing the combining of more complex models (in vivo or 3D organotypic cultures) with very specific cellular measurements. For example, the use of single-cell sequencing can distinguish between genetic changes in the oligodendrocyte population versus changes in other CNS cell types [227,228]. Many other methods, such as imaging (single-cell-resolution microscopy) [229] and single-cell metabolomics [230], can allow for single-cell resolution as well.

## 7. Conclusions

The evaluation of oligodendrocytes and myelin is of great importance, both in developmental neurotoxicity and in studies of demyelinating disorders. Still, there are very few suitable, human-relevant in vitro and in vivo models. It is crucial to further develop and improve upon existing models and assays to study this important cell type. This will allow the improvement of current testing strategies and permit the development of treatments for myelin-related diseases. The development of new models that better mimic human brain physiology has been a priority in recent years, giving rise to next-generation 3D human cell cultures that simulate myelination in vitro. New technologies in stem cells and cell culturing, such as human 3D organotypic models (where it is possible to obtain myelinated axons) [212,213,214,215], are bringing us closer to the in vivo situation of the human brain and have the potential to replace or reduce the use of in vivo and in vitro rodent models. However, these cell cultures also have limitations and associated challenges [220,221,222,223,224,225,226,227,228,229,230,231,232]. It is therefore important to ensure that the application of these models is fit-for-purpose. Drug development for demyelination diseases could clearly be enhanced by these models, to help study the effects of protective compounds, but also the possible mechanisms and interactions with other cell types. In addition, advances in instrumentation, such as microscopy and omics technologies for single-cell resolution and high-throughput screening adaptation, will enable the study of valuable endpoints such as myelin formation.

**Figure 2:**
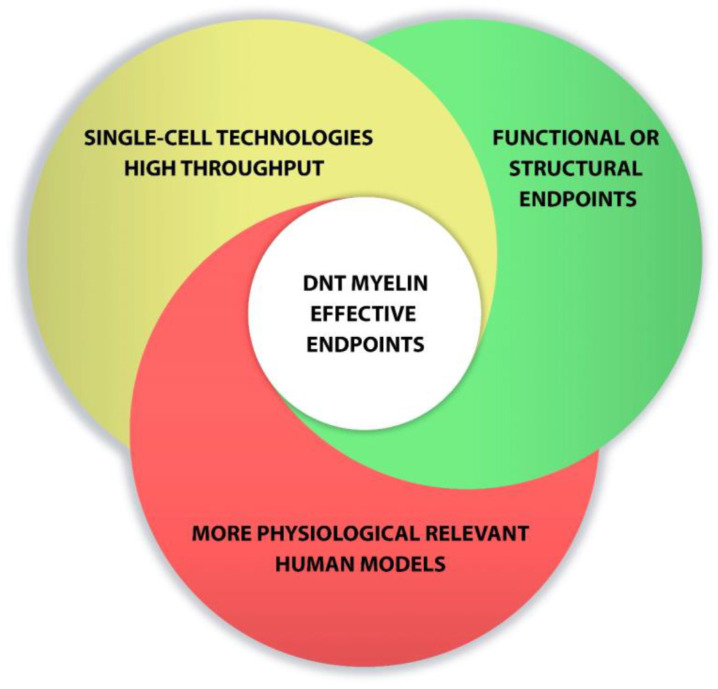
Points to consider for developing DNT myelin effective endpoints.

Overall, new advances in cell culture and the development of new methods to detect 469 developmental neurotoxicity disorders are enhancing progress toward relevant tests to predict human toxicity. Combination of single-cell technologies, high-throughput screening, functional endpoints and the use of more physiological relevant models could favor the development of DNT myelin effective endpoints (Figure 2)
